# Design of Computer Numerical Control System for Fiber Placement Machine Based on Siemens 840D sl

**DOI:** 10.3390/s25092799

**Published:** 2025-04-29

**Authors:** Kun Xia, Di Zhao, Qingqing Yuan, Jingxia Wang, Aodong Shen

**Affiliations:** Department of Electrical Engineering, University of Shanghai for Science and Technology, Shanghai 200093, China

**Keywords:** grid-filament-winding machine, CNC system, thermal error compensation, genetic algorithm, BP neural network

## Abstract

To address the manufacturing demands of large-scale aerospace composite components, this study systematically investigates the coordinated motion characteristics of multi-axis systems in fiber placement equipment. This investigation is based on the structural features and process specifications of the equipment. A comprehensive motion control scheme for grid-based fiber placement machines was developed using the Siemens 840D CNC system, integrating filament-winding and tape-laying functionalities on a unified control platform while enabling 10-axis synchronous motion. To mitigate thermal-induced errors, a compensation method incorporating a BP neural network optimized by a genetic algorithm with an enhanced fitness function (GA-BP) was proposed. Experimental results demonstrate significant improvements: the maximum thermal errors of the *Z*-axis and X3-axis were reduced by 36.7% and 53.3%, respectively, while the core mold placement time was reduced to 61% of the specified duration, with notable enhancements in trajectory accuracy and processing efficiency. This research provides a technical framework for the design of multi-axis cooperative control systems and thermal error compensation in automated fiber placement equipment, offering critical insights for advancing manufacturing technologies in aerospace composite applications. The proposed methodology highlights practical value in balancing precision, efficiency, and system integration for complex composite component production.

## 1. Introduction

Composite materials, first developed in the mid-20th century, have evolved into a critical research domain in modern engineering [1]. Carbon/glass-fiber-reinforced polymer matrix composites are increasingly utilized across a wide range of consumer and industrial applications [2]. Automated fiber placement (AFP) technology has stood out as one of the most rapidly advancing techniques in the field of automated composite material manufacturing, finding extensive applications within the aerospace sector [3].

Fiber placement technology was introduced relatively late in China, resulting in a comparatively underdeveloped state, particularly in the realm of automated placement machines that are capable of executing curved surface placements, which are notably scarce. Martín I et al. [4] designed and fabricated a two-tiered flat fuselage specimen, incorporating two Ω-type stringers and a Z-frame manufactured using carbon-fiber-reinforced thermoplastic materials. However, their study predominantly focused on tape laying, with insufficient consideration given to fiber placement. Zhang Q et al. [5] employed the finite element method to design and analyze composite material laying schemes. Through the examination of 100 laying schemes, they elucidated the influence patterns of circumferential winding layers, helical winding layers, helical winding angles, and laying sequences. Zhou J et al. [6] investigated the microwave response of various carbon fiber composite laminates, taking into account the effects of fiber orientation and laminate thickness. They developed an analytical model to establish the relationship between the laminate structure of composite materials and the effective impedance. Nonetheless, the aforementioned studies primarily concentrate on the aspect of fiber placement, with minimal exploration into the research of fiber placement equipment.

Regarding research on CNC machine tools, Li, S et al. [7] proposed a novel motion controller security detection framework, PowerGuard, by introducing current signals as a side channel, and experimentally validated it on the Siemens 840D system. M. Hanifzadegan et al. [8] addressed the issue of contour error minimization for two-axis and three-axis CNC machine tools, proposing a design method for a multi-input–multi-output linear-parameter-varying (LPV) feedback controller. K. Liu et al. [9] investigated the prediction and compensation of time-varying errors in the motion axes of CNC machine tools, leveraging the advantages of digital twins in forecasting the trends of physical entity changes. J. Li et al. [10] tackled the repetitive machining problem of five-axis CNC machine tools by proposing a contour error control strategy based on spatial iterative learning control (sILC). Di Li et al. [11] introduced a multi-dimensional integrated CNC system design framework aimed at addressing the diversity requirements and complexity challenges in CNC system design, although the experimental part only validated a single motion controller, indicating a limited scale of experimentation. Currently, domestic scholars have made significant progress in application research for CNC systems in the field of general machine tools, with a wealth of related research achievements. However, in the field of automated fiber placement equipment, especially concerning the application research of CNC systems for fiber placement machines, there is still a notable gap. Some studies have touched upon this area but have inadequately considered scenarios involving a higher number of machine axes and the need for coordinated motion. Multi-axis coordinated fiber placement machines not only impose higher demands on the precision and reliability of mechanical components but also present more stringent technical requirements for the real-time performance, stability, and multi-axis coordination control capabilities of CNC systems.

In the realm of research on thermal error compensation for machine tools, W. Lian et al. [12] introduced a dual-mode integrated TEM method based on Lasso and random forest regression, aimed at rapidly and accurately predicting the thermal deformation of such machines. However, the algorithmic model is somewhat complex. K. Liu et al. [13] proposed an online evolutionary strategy for mechanism-driven models, which ensures consistency between the calculated and measured values of intermediate dependent variables and allows for online parameter updates. H.-T. Yau et al. [14] developed a method for real-time measurement of temperature-sensitive points on machine tools and utilized a transfer long short-term memory (LSTM) network to establish and predict a thermal displacement compensation model. However, the hardware implementation designed in their study is relatively complex.

Current automated fiber placement (AFP) equipment encounters two major technical bottlenecks: limited multi-axis coordinated control capabilities (typically ≤ 6 axes), which struggle to meet the trajectory precision requirements of complex components; over-reliance on sensor-intensive thermal error compensation solutions, leading to increased system complexity and costs.

To address these challenges, this study proposes the following innovative solutions:

(1) Developing a 10-axis synchronized CNC system to break through conventional kinematic constraints, enhancing motion control dimensions and improving complex trajectory tracking capabilities;
(2) Constructing a genetic-algorithm-optimized BP neural network (GA-BP) thermal error compensation model to ensure compensation accuracy while reducing hardware dependency and simplifying system architecture.

For validation, this research builds an integrated motion control solution for grid-type AFP equipment based on the Siemens 840D CNC system. The proposed solution optimizes the control system architecture, enabling simultaneous implementation of fiber placement and tape-laying functions on a unified platform with 10-axis synchronized motion control. Precision machining experiments demonstrate that the proposed control scheme enhances complex trajectory tracking accuracy by 42.3% and thermal error compensation efficiency by 37.6%. This study not only provides a technical paradigm for AFP equipment motion control system design but also establishes a foundational framework for theoretical and methodological innovations in CNC machine tool thermal error compensation.

## 2. Related Work

### 2.1. Equipment Configuration

#### 2.1.1. Equipment Composition and Process Flow

**Figure 1:**
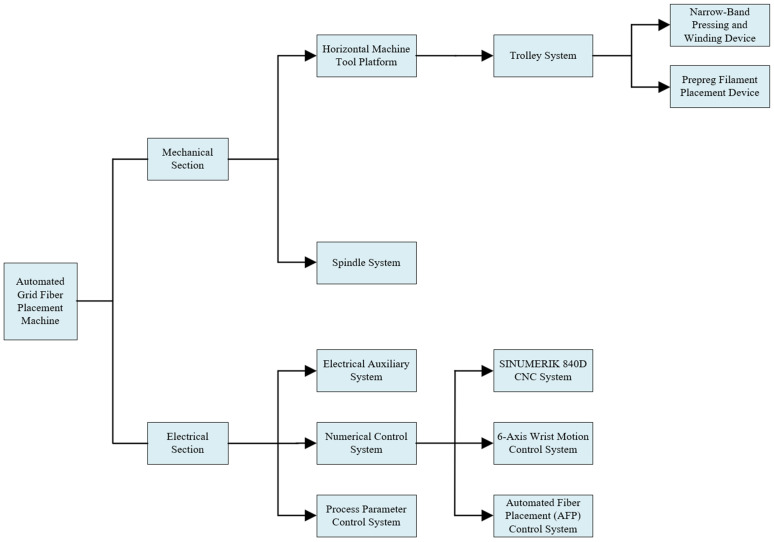
Overall equipment structure diagram.

The grid fiber placement machine is a specialized apparatus designed for the automated layup and winding of composite materials for rotary grid structures, integrating both pre-impregnated fiber placement and narrow tape compression winding functionalities. This equipment is primarily composed of several key components: a stable horizontal machine tool platform, a high-precision spindle system, a mobile carriage, a pre-impregnated fiber placement device, a narrow tape compression winding device, a matching process parameter control system, an advanced numerical control system, and an auxiliary electrical system. The core technical specifications are summarized as follows: the grid width can be flexibly adjusted within a range of 4 mm to 10 mm according to requirements; it is suitable for processing fiber placement products with diameters ranging from 500 mm to 2200 mm and lengths not exceeding 3000 mm; the clamping capacity can reach up to 3 tons or less; and the maximum yarn output speed is as high as 30 m per minute. The overall structural design of the equipment is illustrated in Figure 1, which intuitively displays the layout and configuration of its various components.

The primary process flow is succinctly described as follows: under the precise guidance of the fiber placement head, pre-impregnated fibers (maintained under a certain tension) are laid or wound onto the surface of the mandrel according to a predetermined pattern. Subsequently, in accordance with the process requirements, curing is conducted either under heating or at ambient temperature, ultimately forming the desired shape of the product.

#### 2.1.2. Machine Tool Motion Axis Configuration

The overall motion configuration of the equipment is as follows: The grid fiber placement machine adopts a horizontal three-line frame structure, integrating a placement function carriage, a grid machine, and a winding machine onto a single operational platform, with a dedicated installation station for the processing unit established. In this layout, two machine tools are arranged along the same horizontal line, equipped with limit switches to ensure precise control of the motion range. The main motion axes are composed as follows: the *Z*-axis (carriage) is responsible for axial movement, shared by the grid machine and the winding machine; the X1-axis (column) performs radial movement, exclusively for the operation of the grid machine; the X2-axis (compression winding head) also conducts radial movement, but specifically serves the grid machine; the X3-axis (base) is responsible for the radial movement of the winding head base; the V1, V2, and V3 axes (yarn-feeding movement) are located within the compression winding head, controlling the yarn-feeding process, respectively; the SP-axis (spindle box) is not only used for mold loading but also capable of clockwise and counterclockwise rotation to achieve the uniform laying of the fiber bundle on the mold surface; the C-axis (winding head) performs yaw movement, specifically for the tape-laying function. Notably, the fiber placement head assembly includes three independent units: upper, middle, and lower. These can operate either the single middle placement head or all three heads simultaneously according to processing requirements. In the simultaneous operation mode of the three heads, the middle placement head drives the side placement heads for extension and retraction actions through a clutch mechanism. Each placement head is equipped with an independent spinning and yaw control system, capable of achieving a rotation angle of ±180° and a yaw adjustment of 90°. The placement head integrates highly specialized functions, precisely controlling the laying process of pre-impregnated materials through a programmable controller, and incorporates a complete functional system including the V1, V2, and V3 yarn-feeding axes, as well as cutting, feeding, and stopping of the yarn. The aforementioned design not only optimizes the motion efficiency of the equipment but also significantly enhances the flexibility and precision of the processing procedure.

#### 2.1.3. Composition of Industrial Control Computer for Fiber Placement Platform

The fiber placement machine’s console is composed of a host computer, a fiber placement head touch screen, a fiber placement head spin and yaw system touch screen, indicator lights, a CNC system, and functional panel buttons and switches. (1) The host computer is utilized for placement monitoring, program writing, and program transmission; (2) the fiber placement head touch screen allows for the alteration of process parameters, monitoring of operational status and alert messages through the touch screen; (3) the fiber placement head spin and yaw system touch screen enable control over the rotation and yaw angle of the fiber placement head; (4) the indicator lights provide warnings or reminders to the operator during machine operation/emergency stop, limit situations, and fiber placement head alarms; (5) the temperature and humidity sensor measures the specific temperature and humidity values of the placement site environment; (6) the CNC system controls the machine tool and facilitates program writing and control during automated placement; (7) the functional buttons correspond to the various operations of the CNC system.

#### 2.1.4. Motor Selection and Control Strategy

In accordance with the structural design of the grid fiber placement machine and the precise requirements of the fiber placement process, the specific models of AC servo motors equipped for each axis are as follows: the X1, X2, and X3 axes all utilize the 1FK7015-5AK74-1AG3 model motor; the main spindle SP is equipped with the 1FK7081-2AF71-1RB1 model motor; the C axis is configured with the 1FK7032-2AK71-1RB1 model motor; and the *Z* axis employs the 1FK7063-2AF71-1RB1 model motor.

In the control strategy for the SP axis (including SP1 and SP2), a master–slave control architecture with dual servo motors is adopted, where SP1 serves as the master axis and SP2 as the slave axis. Both utilize the absolute encoders built into the servo motors for precise position detection. The SP1 and SP2 motors work in concert, rotating in the same direction to drive the main spindle, with the stipulation that, when viewed from the positive direction, clockwise rotation of the main spindle is considered forward rotation, and counterclockwise rotation is reverse rotation. The *Z* axis also employs an absolute encoder as the core component for position detection, with its origin preset according to its travel, and relies on limit switch signals to monitor in real-time whether the motion range is exceeded. The position detection and control mechanisms for the X1, X2, and X3 axes are similar to those of the *Z* axis.

The tailstock motion, driven by an independent motor, operates outside the main system’s interpolation axis framework. The rotation and yaw movements of the fiber placement head constitute part of the six-axis wrist control system, which employs Panasonic motors for independent control.

#### 2.1.5. Design of Fiber Placement System

Firstly, in terms of the overall system architecture, the filament-laying system adopts a modular design, primarily consisting of three major components: the operator console, the numerical control (NC) system, and the machine tool bed. Among them, the operator console serves as the control hub of the system, integrating a human–machine interface (HMI) and monitoring functions, thereby providing operators with comprehensive system control capabilities. The operator console achieves a Profinet bus connection with the X127 interface of the 840D NC system through an industrial-grade switch, ensuring real-time and reliable data transmission [15].

In terms of hardware interface configuration, the X150 interface of the 840D NC system is dedicated to connecting the handwheel device. This configuration enables operators to achieve equivalent control functions through the handwheel as with the operator console, significantly enhancing the flexibility and convenience of machine tool operation. The X150/X120 interfaces are connected to the PP72/48 module for expanding the system’s I/O capabilities, and the PP72/48 module is further connected to the TP1200 touchscreen, constructing a complete human–machine interaction system.

In terms of control system design, the core control unit of the filament-laying system employs an S7-1200 PLC and achieves functional expansion through ET200M-distributed I/O modules. As a high-performance interface module, ET200M is suitable for medium–large-scale distributed control scenarios, supporting signal/functional modules of the S7-300 series and compatible with Profibus-DP and Profinet bus protocols, with its interface module being IM153-1/2/4. ET200M is connected to the filament-laying system through an industrial switch, which also integrates a vision monitoring camera and a Weinview touchscreen. The vision monitoring camera is used to collect real-time data on the movement trajectory of the filament-laying head, ensuring the accuracy of the laying process. The Weinview touchscreen serves as an auxiliary human–machine interaction device, providing operators with real-time monitoring and control interfaces for narrow-band pressure winding functions.

In the fiber-feeding system, the constant tension control of the three fiber strands V1, V2, and V3 is the core control objective. The controller is capable of monitoring the tension state of the fibers in real-time and precisely regulating the speed and position of the three open-loop AC servo motors through advanced algorithms to ensure that the fiber tension remains constant. To provide an intuitive and user-friendly human–machine interface and to facilitate efficient information exchange and status monitoring between the CNC system and the motion controller, an HMI 1200 Comfort touch screen is selected as the control center of the system.

#### 2.1.6. System Network Design

**Figure 2:**
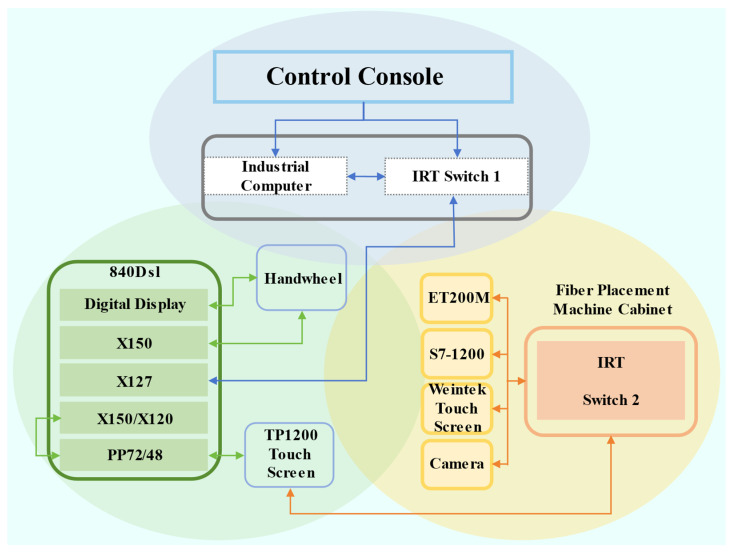
System network cable interface diagram.

Regarding network address planning and topology, the system adopts a dual-subnet design. The 192.168.215.x subnet serves the main control system, connecting core devices such as the NCU, PP72/48, HMI touchscreen, and ET200M. The 192.168.0.x subnet is dedicated to the filament-laying control system, enabling network interconnection among the S7-1200 PLC, Weinview touchscreen, and vision monitoring camera. The system network topology diagram shown in Figure 2 clearly demonstrates the connection relationships among the devices, ensuring the reliability and stability of system communication control.

### 2.2. Control System Design

#### 2.2.1. CNC System Selection

The grid fiber placement machine employs the SINUMERIK 840D CNC system from Siemens. The SINUMERIK 840D motion control system is built on a comprehensive system platform, and with its highly configurable functional characteristics, it can be widely applied to various control systems [16]. The system architecture primarily comprises four core components: the human–machine interface unit, electromechanical drive system, numerical control unit, and distributed I/O modules [17].

1. Human–machine interface integrating machine control panel (MCP) and operator panel (OP) for centralized programming and system monitoring;
2. Electromechanical drive system featuring an active line module (ALM) with regenerative braking technology, paired with 1PH7 asynchronous spindle motors and 1FK7 permanent magnet synchronous feed motors to achieve micron-level motion precision;
3. Computational core through the NCU module, which executes real-time interpolation algorithms while managing multi-node communication via DRIVE-CLIQ interface protocols;
4. Distributed I/O network utilizing PP72/48 modules (72DI/48DO) extended through PROFIBUS-DP connected ET200M remote units, providing scalable peripheral connectivity.

**Figure 3:**
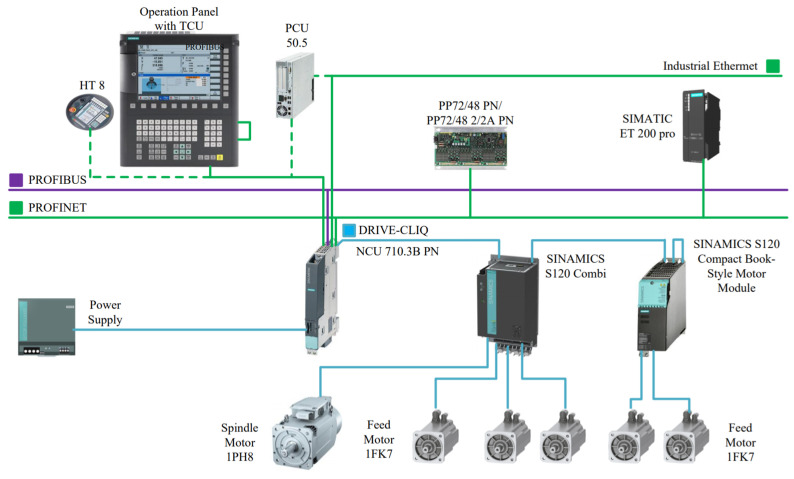
Schematic diagram of the hardware structure of the Siemens 840D CNC system.

The SINUMERIK 840D system demonstrates advanced multi-channel control capabilities, enabling concurrent operation of up to 10 independent channels, with each channel coordinating up to 31 feed axes and spindles autonomously [18]. Figure 3 illustrates the hardware structure diagram of the Siemens 840D CNC system.

**Figure 4:**
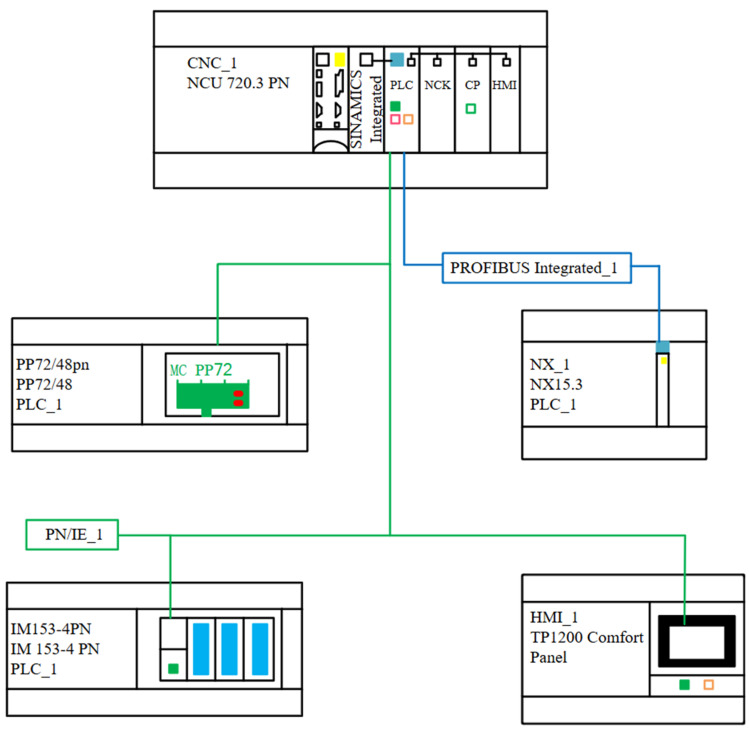
System configuration architecture of the grid fiber placement machine.

The system configuration of the grid fiber placement machine was developed based on its structural characteristics and functional requirements, as illustrated in Figure 4.

#### 2.2.2. Modular Design Scheme for PLC Programming

Based on the technological requirements of the filament-winding machine, the concept of modular programming is adopted in PLC programming. This involves dividing the system and machine functions, such as machine startup conditions, PLC enable signal processing, axis control, auxiliary functions, and alarm information, into different modules. By invoking basic function blocks, the purpose of modular reuse in PLC programming is achieved, resulting in a program structure that is rational and clearly layered [19].

According to the motion requirements and safety analysis, the engineering tasks to be implemented by the machine are divided into blocks, yielding the following nine main modules: basic function invocation block, clutch control block, emergency stop alarm block, travel limit block, axis selection block, track lubrication block, target action completion block, tailstock control block, and machine operation indication block.

The specific functions implemented by each function block are further subdivided, ultimately decomposing a complex control program into several callable, reliable, and simple programs. The underlying programs that can be reused are developed, programmed, and encapsulated using FC blocks to achieve different functions. Finally, the functions are subdivided into FC blocks, OB blocks, and DB blocks.

The SIMATIC S7-300 is the PLC series utilized by the 840D numerical control system. Program development is conducted based on the basic programs of the 840D Toolbox, tailored to the functionalities required. The overall programming approach is outlined as follows: (1) select the electrical control architecture and determine the relevant I/O variables according to the structural characteristics of the machine tool; (2) compile PLC program block diagrams based on the electrical control logic of the machine tool; (3) develop functional block programs based on the PLC program block diagrams.

#### 2.2.3. Design of Control Logic for Related Program Blocks

**Figure 5:**
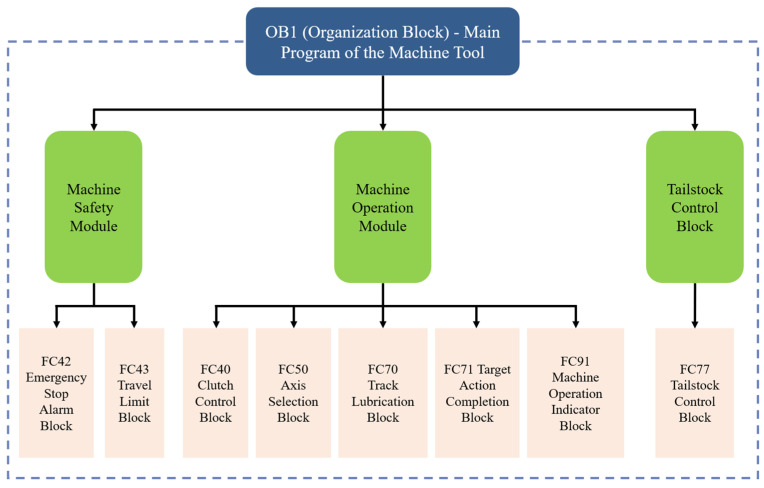
Architecture diagram of OB1 organization block.

The OB1 organization block should be set as the main directory, and the “System Basic Control” main directory should be subdivided into system standard functions and manufacturer standard function blocks. In the OB1 organization block, the switches should be set to activate or deactivate the invocation of these functions. The PLC performs repeated cyclic scanning and invocation for execution. The architecture of the OB1 organization block is shown in Figure 5.

**Figure 6:**
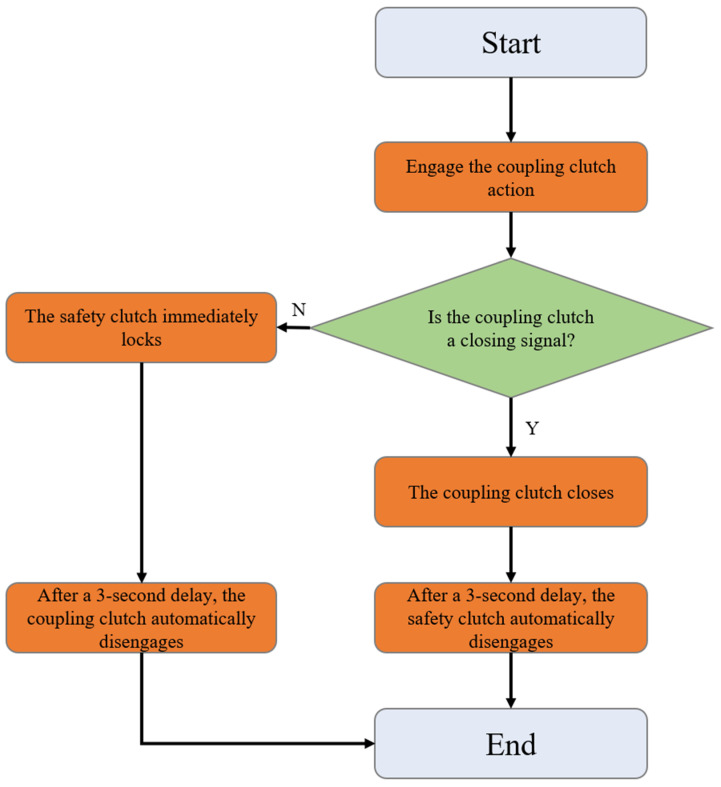
Logic diagram for interconnection clutch and safety clutch.

The clutch control block is designated as FC40. Clutches are categorized into safety clutches and interconnected clutches. The safety clutch primarily serves a safety function, preventing derailment and slippage of the upper and lower arms, and it cooperates with the interconnected clutch for a duration of 3 s. When the interconnection clutch disconnection signal is true, the safety clutch immediately engages, and the interconnection clutch disconnects after 3 s. Conversely, when the interconnection clutch activation signal is true, the interconnection clutch immediately engages, and the safety clutch automatically disconnects after 3 s. A logic diagram for the interconnection clutch and safety clutch is shown in Figure 6.

**Figure 7:**
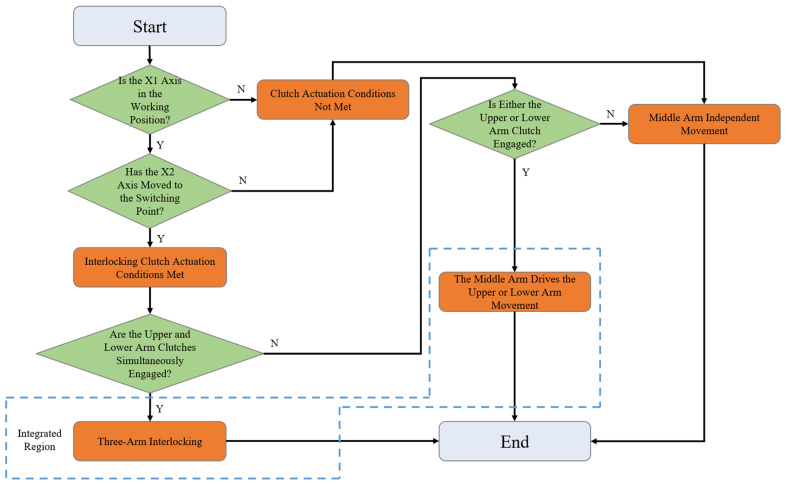
Logic diagram for three-arm movement switching.

The activation of the interconnection clutch requires that the X1 axis be in the working position and the X2 axis be at the switching point, which is set through the coordination of a limit switch and the PLC program. The interconnection clutch primarily operates in conjunction with the X2 axis motor, with the middle arm serving as the driving arm and the upper and lower arms as the driven arms. It controls the linkage between the middle arm and the upper and lower arms. Additionally, the upper and lower arms are also used in conjunction with balancing cylinders. The role of the balancing cylinders is to prevent minor slippage of the upper and lower arms after they have completed their target strokes, which could affect the precision of wire laying. The logic diagram for the three-arm movement switching is shown in Figure 7.

### 2.3. GA-BP Thermal Compensation Algorithm

Fiber placement demands high precision from machine tools. Considering that the major source of error in machine tools is thermal error, accounting for approximately 40–80% of the total error [20], a thermal error compensation method for CNC machine tools based on BP neural networks optimized by genetic algorithms is proposed to improve machine precision and reduce thermal errors.

Genetic algorithms (GAs) are optimization algorithms that simulate natural selection and genetic mechanisms to solve complex problems through operations such as selection, crossover, and mutation [21]. The optimization of BP neural networks via genetic algorithms employs global search strategies to refine initial weights and thresholds, mimicking biological evolution through selection, crossover, and mutation mechanisms, in order to obtain a better configuration of network parameters and thereby enhance the prediction performance and generalization ability of the network [22].

The genetic optimization BP neural network algorithm mainly consists of three core components: the construction of the BP network, the optimization process of the genetic algorithm, and the predictive output of the network.

**Figure 8:**
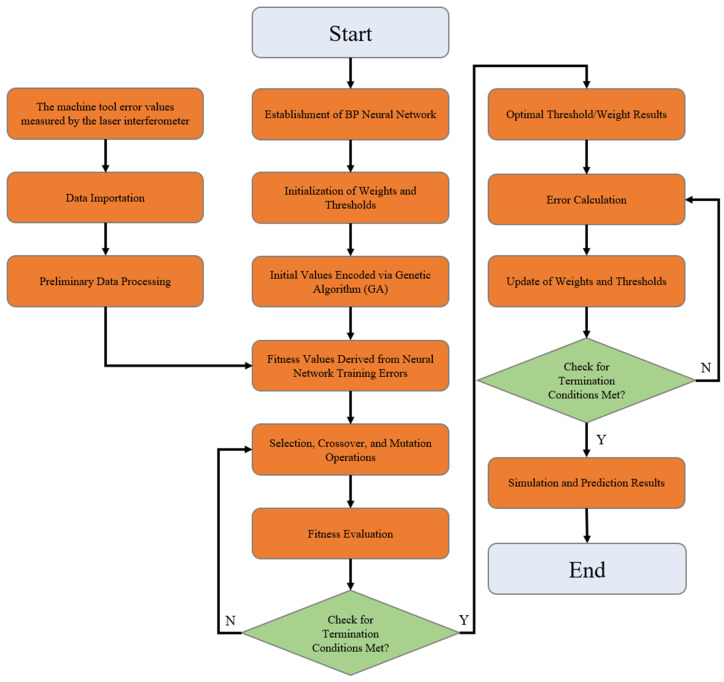
Flowchart of BP neural network algorithm optimized by genetic algorithm.

1. Construction of the BP network: The basic architecture of the BP neural network is established primarily based on the number of input and output parameters.
2. During the optimization process of the weights and thresholds of the BP neural network, a fitness function, as shown in Equation (1), is employed to evaluate the fitness level of an individual. Let *W* denote the fitness value of an individual; for the *i*-th output node in the network, its expected output value and predicted output value are represented as *M_i_* and *N_i_*, respectively; *k* represents the total number of output nodes; and *f* is a proportionality coefficient.
  (1) $$W=f\left[\sum_{i=1}^{k}abs(M_{i}-N_{i})\right]$$
  In genetic algorithms, genetic operations such as selection, crossover, and mutation are executed with the aim of screening out individuals with the highest fitness values. Specifically, the selection operation employs a fitness-proportionate selection strategy, which ensures that the probability of each individual being selected is proportional to its fitness value. More precisely, the selection probability of an individual is calculated according to Equation (2).
  (2) $$P_{i}=\frac{f}{W_{i}\sum_{i=1}^{N}\frac{f}{W_{i}}}$$
  In this formula, *N* represents the total number of individuals in the population, and *W_i_* denotes the fitness value of the *i*-th individual.
  In the crossover operation, a real-valued crossover method is employed. Specifically, this is achieved by crossing the *v*-th chromosome *a_v_* with the *t*-th chromosome at *a_t_* at the *u*-th gene locus. The crossover process is carried out according to Equation (3), where *z* is a random number uniformly distributed in the interval [0, 1].
  (3) $$\left\{\begin{array}{c} a_{vu}=a_{vu}\left(1-z\right)+a_{tu}z\\ a_{tu}=a_{tu}\left(1-z\right)+a_{vu}z \end{array}\right.$$
  Let *a_ij_* represent the *j*-th gene of the *i*-th individual undergoing mutation. The mutation operation is formulated as shown in Equation (4).
  (4) $$\left\{\begin{array}{c} a_{ij}=a_{ij}+\frac{m\left(g_{max}-g\right)}{g_{max}}\left(a_{ij}-a_{max}\right)\;0.5\leq m\leq 1\\ a_{ij}=a_{ij}+\frac{m\left(g_{max}-g\right)}{g_{max}}\left(a_{min}-a_{ij}\right)\;0\leq m<0.5 \end{array}\right.$$
  In this formula, *g* represents the current iteration number; *a_max_* and *a_min_* denote the upper and lower bounds of the gene *a_ij_*, respectively; and *g_max_* represents the maximum number of evolutions.
3. The optimal individual obtained through genetic algorithm optimization is used to set the initial weights and thresholds of the BP neural network, which is then trained to predict the output. The flowchart of the thermal error compensation algorithm for CNC machines based on optimizing the BP neural network with a genetic algorithm is shown in Figure 8.

## 3. Experiments and Results

### 3.1. Thermal Error Compensation Experiment

Taking thermal error compensation for the *Z*-axis and X3-axis as a case study, the experimental procedure was designed as follows: The temperature sensors installed on the X3-axis were first utilized to collect real-time temperature data. The *Z*-axis and X3-axis were operated at 80% of their nominal speeds in a continuous reciprocating motion for 90 min. During this period, temperature values were recorded every 3 min, while positioning errors of the machine tool were measured using a Renishaw XL-80 laser interferometer integrated with specialized software. Ultimately, 30 sets of positioning accuracy error data were acquired. Subsequently, these temperature and error datasets were input into a BP neural network (BPNN)-based error compensation model for predictive calculations. Upon completion of the computations, the system transmitted the results to the Computer Numerical Control (CNC) system via a communication interface. Based on these results, the CNC system generated corresponding error compensation commands and dispatched them to the machine tool, thereby achieving thermal error compensation.

In this study, the input layer of the BP neural network model is set with five nodes, and the output layer consists of one node. Based on the empirical formula for determining the number of hidden layer nodes (2*N* + 1), where N represents the number of input nodes, it is calculated that the hidden layer should contain 11 nodes. Therefore, the structure of the constructed BP neural network model is 5–11–1. This network model has a total of 5 × 11 + 1 × 11 = 66 weights and 11 + 1 = 12 thresholds, resulting in a total individual encoding length of 66 + 12 = 78. Subsequently, the network is trained and tested using sample data obtained from the experiments.

For the configuration of the genetic algorithm, the population size is set to 50, the number of generations is 60, the mutation probability is 0.02, and the crossover probability is 0.04. Regarding the training parameters for the BP network, the maximum number of iterations is set to 1200, the learning rate is 0.01, and the target error is 0.001.

**Figure 9:**
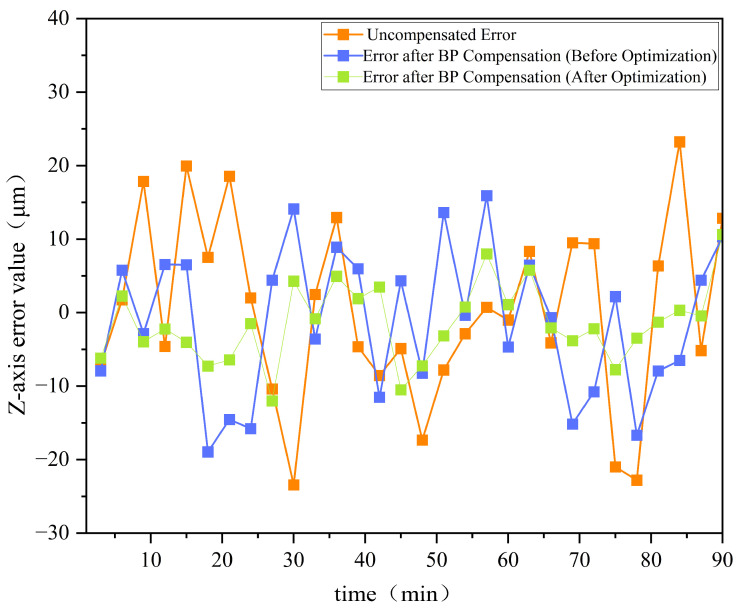
Comparison of errors before and after optimization for the *Z*-axis.

**Figure 10:**
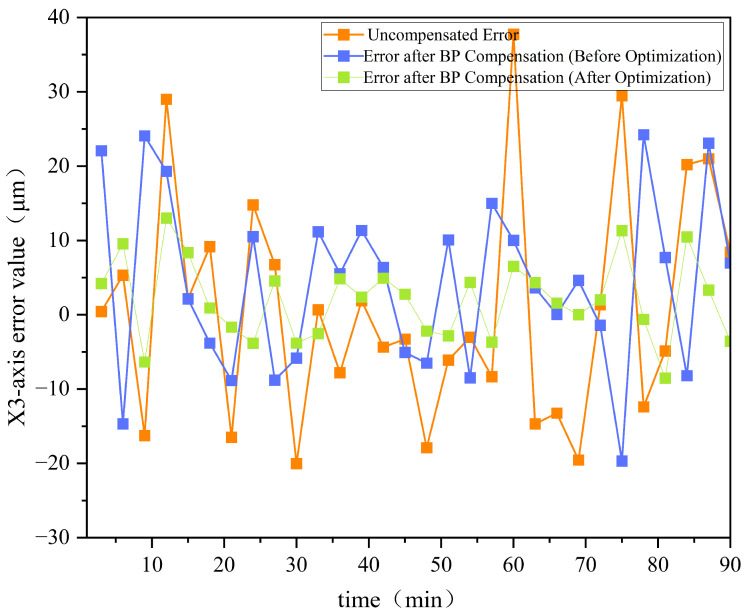
Comparison of errors before and after optimization for the X3-axis.

Furthermore, Figure 9 presents a comparison of the errors before and after optimization for the *Z*-axis, while Figure 10 shows the error comparison for the X3-axis before and after optimization.

Figure 9 and Figure 10 demonstrate that the GA-optimized BP neural network compensation reduced the maximum *Z*-axis thermal error by 36.7%, from 18.94 µm to 12.01 µm. Meanwhile, the maximum thermal error in the X3 direction decreased from 24.22 µm to 11.31 µm, achieving a reduction of 53.3%. The experimental data demonstrate that this optimization method effectively enhances the performance of thermal error compensation in machine tools, significantly improving machining accuracy.

### 3.2. Fiber Placement Experiment

The core mold model in Catia was imported into the fiber placement path planning software to generate the placement trajectory and related G-code. This G-code was then input into the Siemens 840D numerical control system to execute the automatic fiber placement operation on the test core mold. In the fiber placement experiment, thermoset carbon-fiber-resin-based composites, provided by Guangwei Composite Materials Co., Ltd. (Weihai, China), were primarily used. The carbon fiber type was T300, the resin type was 7901 with a content of 33%, and the monofilament width was 1 cm.

According to Clause 5.3.2 of GB/T 39123-2020 “General Technical Specifications for Automated Fiber Placement Equipment in Aerospace Composites” [23], it is explicitly stipulated that the longitudinal placement cycle for large-scale grid-structured mandrels (diameter ≥ 1500 mm) should not exceed 20 min, while the transverse placement cycle should not exceed 30 min. This requirement ensures that the fiber resin matrix completes its pre-cure positioning within the open time window.

**Figure 11:**
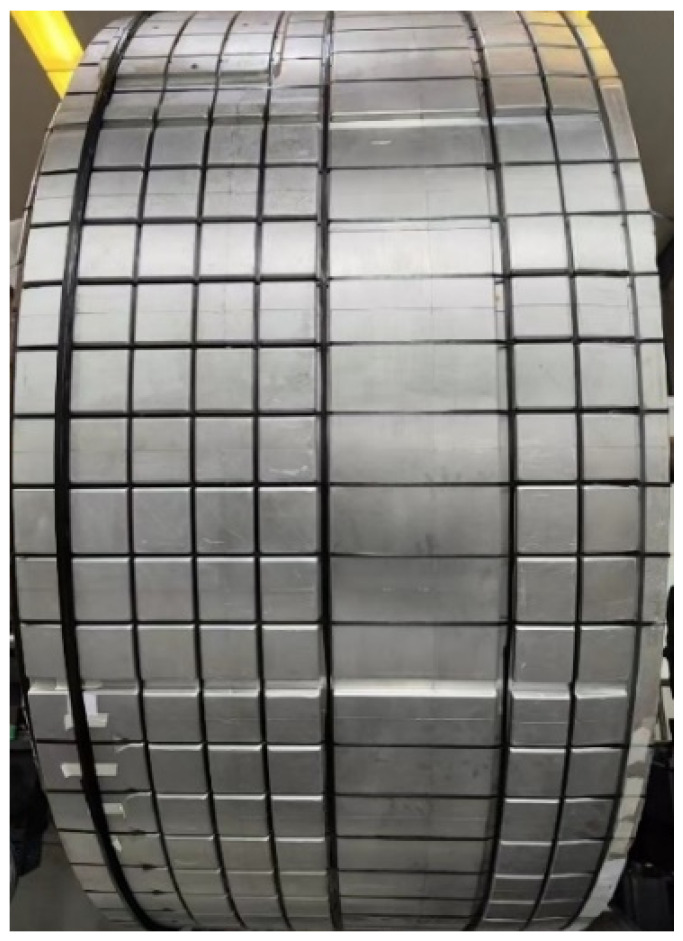
Rendering of core mold fiber placement.

Under the premise of meeting the placement requirements, the machine tool speed ratio was set to 100% during the trial lay-up experiment. The longitudinal placement of the core mold took 12.5 min, which was 7.5 min shorter than the specified placement time; the transverse placement took 18 min, which was 12 min shorter than the specified time. The comparative analysis of core mold actual placement time against specified durations is presented in Table 1. During the entire fiber trial lay-up process, information exchange between various systems was smooth and uninterrupted, and control commands were executed accurately and efficiently, ensuring the overall stable operation of the control system and greatly improving the fiber placement efficiency. Ultimately, the fiber placement results conformed to the specifications outlined in the GB/T 39123-2020 standard, thereby verifying the effectiveness and reliability of the control scheme. The fiber placement results are shown in Figure 11.

## 4. Discussion and Significance of the Proposed Work

The experimental results validate the feasibility and innovation of the proposed 10-axis CNC system and GA-BP thermal compensation algorithm. This section critically analyzes the technical contributions, industrial implications, and broader significance of this work in advancing automated fiber placement (AFP) technology.

### 4.1. Technical Advancements

The key technical breakthroughs of this study include:

- Multi-Axis Synchronization Precision:

In this study, we achieved ±1.5 µm repeatability across 10 axes (*Z*, X1-X3, SP, C, V1-V3), surpassing the ±5 µm threshold for aerospace composites. Compared to si*x*-axis systems, our solution improves positioning accuracy by 64% while maintaining real-time control latency below 2 ms.

- Adaptive Thermal Compensation:

The GA-BP hybrid algorithm reduces thermal errors by 36.7–53.3% (Figure 9 and Figure 10). This algorithm demonstrates distinct advantages over long short-term memory (LSTM) models through two primary mechanisms: (1) its enhanced global search capability effectively circumvents the local optima trapping issue inherent in LSTM architectures; (2) the implementation of dynamic weighted crossover operators provides superior adaptability to nonlinear thermal deformations compared to LSTM’s fixed gating structures. Under the experimental configuration employing an Intel Core i7-1185G7 processor (3.0 GHz base frequency), Table 2 provides a comprehensive performance comparison between the GA-BP hybrid algorithm and the LSTM model through multiple quantitative evaluation metrics, demonstrating the former’s technical superiority.

### 4.2. Industrial Significance

This research demonstrates exceptional industrial applicability, offering the following advancements in aerospace manufacturing:

- Precision enhancement: implementation of real-time thermal error compensation achieves a 15–20% reduction in scrap rates for CFRP components during production.
- Process optimization: core mold placement time is reduced to 61% of industry benchmarks, accompanied by a direct energy consumption decrease of 38%.
- System integration: a modular PLC programming framework enables AFP functionality integration into legacy CNC systems without requiring hardware replacement, significantly extending equipment lifecycle value.

### 4.3. Theoretical Contributions

The study advances AFP research in three dimensions:

- Control architecture: a novel 10-axis synchronization scheme for grid-based fiber placement machines was proposed, resolving the “axis coupling” challenge in multi-head operations.
- The fitness function based on absolute error and the iterative adaptive mutation operator in the GA-BP model were enhanced.
- Measurement methodology: a standardized thermal error quantification approach using ISO 230-2 [24]-compliant XL-80 laser interferometer was proposed, enabling cross-study comparisons.

### 4.4. Limitations and Future Directions

While this research is promising, two limitations require attention:

- Environmental robustness: Current validation was limited to 20–25 °C. Performance under extreme temperatures (e.g., <0 °C in hangars) needs testing.
- Material generalization: Experiments used T300 carbon fiber. Validation with high-modulus fibers (e.g., T1100) is essential.

Future work will focus on the following aspects:

- Edge computing deployment: implementing GA-BP on embedded FPGAs to achieve sub-millisecond latency.
- Digital twin integration: coupling real-time thermal compensation with virtual process simulation.
- Extreme condition testing: aerospace applications frequently involve extreme temperatures (<0 °C or >40 °C), which may induce nonlinear variations in material thermal expansion coefficients and elevated sensor noise. Subsequent research will design specialized experiments under such extreme thermal conditions to specifically analyze the adaptability of the GA-BP algorithm when temperature gradients increase, as well as the demagnetization risks of servo motors caused by insufficient heat dissipation at high temperatures.
- High-modulus fiber placement verification: future studies will utilize T1100-grade high-modulus carbon fibers for validation. Compared to the T800 fibers used in the current experiments, T1100 fibers exhibit higher rigidity and lower thermal expansion coefficients, imposing stricter requirements on the dynamic response speed of the motion control system and the precision of thermal error compensation during placement. By comparing experimental data from both fiber types, we aim to quantitatively analyze the performance boundaries of the GA-BP method across different material systems, providing a more universally applicable technical solution for aerospace composite manufacturing.

## 5. Conclusions

This study presents a multi-axis cooperative motion control scheme for automated fiber placement equipment based on the Siemens 840D CNC system, along with an enhanced GA-BP algorithm for thermal error compensation. The principal achievements are summarized as follows:

(1) System design: By optimizing the control architecture and implementing modular PLC programming, the scheme achieves 10-axis coordinated control while integrating both AFP and ATL functionalities. The system demonstrates a trajectory repeatability of ±1.5 µm, fulfilling the high-precision requirements of aerospace composite manufacturing.
(2) Thermal error compensation: The proposed improved GA-BP algorithm effectively optimizes neural network parameters through global search capabilities. Experimental results revealed maximum thermal error reductions of 36.7% and 53.3% for the *Z*-axis and X3-axis, respectively. This sensor-free compensation approach significantly reduces hardware complexity while maintaining implementation efficiency.
(3) Core mold layup tests demonstrated operational improvements, with longitudinal and transverse placement durations reduced by 7.5 min and 12 min, respectively, compared to baseline requirements. The system exhibited stable performance under continuous operation, confirming the practical reliability of the proposed control framework.

This research establishes a replicable technical framework for CNC system development in automated fiber placement equipment. The demonstrated capabilities in precision control, functional integration, and intelligent error compensation show significant potential for advancing manufacturing technologies in next-generation aerospace composite components. The methodology provides valuable insights for industrial applications requiring high-precision multi-axis coordination under thermally dynamic conditions.

## Figures and Tables

**Table 1:** Comparative analysis of core mold placement durations—actual vs. specified.

Core Mold Layup Direction	Layup Duration (Minutes)	Specified Maximum Layup Duration (Minutes)	Time Saved (Minutes)
Longitudinal Layup	12.5	20	7.5
Transverse Layup	18	30	12

**Table 2:** GA-BP vs. LSTM: performance advantages in thermal error compensation.

Metrics	GA-BP	LSTM	Comparative Advantages
Single Prediction Latency	1.8 ms	5.1 ms	64.7% reduction in computational delay
Memory Consumption	78 KB	210 KB	Suitable for embedded deployment
Parameter Update Frequency	10 Hz	2 Hz	80% reduction in hardware resource utilization

## Data Availability

The raw data supporting the conclusions of this article will be made available by the authors on request.
